# Suture of the Extensor Tendons of the Fingers, with a Case of Cure by This Treatment

**Published:** 1858-11

**Authors:** 


### ECLECTIC DEPARTMENT,

### AND SPIRIT OF THE MEDICAL PERIODICAL PRESS.

---

### *Suture of the Extensor Tendons of the Fingers, with a Case of Cure by this Treatment.* By M. Mourgue.

Suture of the small tendons, like the extensors and flexors of the fingers, is a triumph of modern surgery; and the happy results which have followed its use have given it a place among legitimate operations. The case of M. Mourgue adds another instance of success to those already recorded.

Case. A maker of wooden shoes received on the back of his left hand, on the 10th of December, a blow of a hatchet, which divided the extensor tendons of the fore and middle finger at the metacarpo-phalangian joint. The lower ends presented at the wound, but the upper were retracted beneath the skin to the extent of nearly an inch. An incision carried up to the ends of the retracted tendons, allowed them to be seized by forceps and pierced by a needle armed with a waxed thread; this needle was then passed through the corresponding ends of the tendons below, and they were thus brought into contact with those above, and tied. The external wound was also closed with sutures. The hand was extended on a wide, flat splint, and the wound covered with a linen bandage spread with cerate, and compresses wet with cold water.

12th. There is considerable swelling of the wrist and great redness; it is necessary to remove the sutures from the wound. In a few days, the inflammation subsided.

20th. The wound, which is open, but free from redness and inflammation, is in good condition. The edges were brought together with sticking-plaster.

The ligatures of the tendons came away on the 24th and 26th; the external wounds cicatrized at once. On the 8th of January, the splint was dispensed with.

Jan. 22d. The man has resumed his work, the fingers having gradually recovered their strength and mobility, and having complete power of extension and flexion. In a word, the suture of the extensor tendons has been attended with all the success which could be desired.—*Gazette Médicale,* from *Boston Med. and Surg. Journal.*

